# Early detection of deteriorating patients in general wards through continuous contactless vital signs monitoring

**DOI:** 10.3389/fmedt.2024.1436034

**Published:** 2024-08-29

**Authors:** Ambuj Yadav, Himanshu Dandu, Gaurav Parchani, Kumar Chokalingam, Pooja Kadambi, Rajesh Mishra, Ahsina Jahan, Jean-Louis Teboul, Jos M. Latour

**Affiliations:** ^1^ Department of Medicine, King George's Medical University, Lucknow, India; ^2^ Department of Clinical Research, Turtle Shell Technologies Private Limited, Bengaluru, India; ^3^ Paris-Saclay Medical School, Paris-Saclay University, Le Kremlin-Bicêtre, France; ^4^ Faculty of Health, University of Plymouth, Plymouth, United Kingdom

**Keywords:** Early Warning System (EWS), remote, contactless, continuous, monitoring, general ward, ballistocardiography

## Abstract

**Objective:**

To assess the efficacy of continuous contactless vital signs monitoring with an automated Early Warning System (EWS) in detecting clinical deterioration among patients in general wards.

**Methods:**

A prospective observational cohort study was conducted in the medical unit of a tertiary care hospital in India, involving 706 patients over 84,448 monitoring hours. The study used a contactless ballistocardiography system (Dozee system) to continuously monitor heart rate, respiratory rate, and blood pressure. The study assessed total, mean, and median alerts at 24, 48, 72, 96, 120 h, and length of stay (LOS) before patient deterioration or discharge. It analyzed alert sensitivity and specificity, average time from initial alert to deterioration, and healthcare practitioners (HCP) activity. Study was registered with the Clinical Trials Registry-India CTRI/2022/10/046404.

**Results:**

Out of 706 patients, 33 (5%) experienced clinical deterioration, while 673 (95%) did not. The deterioration group consistently had a higher number of alerts compared to those who were discharged normally, across all time-points. On average, the time between the initial alert and clinical deterioration was 16 h within the last 24 h preceding the event. The sensitivity of the Dozee-EWS varied between 67% and 94%. HCP spend 10% of their time on vital signs check and documentation.

**Conclusions:**

This study suggests that utilizing contactless continuous vital signs monitoring with Dozee-EWS in general ward holds promise for enhancing the early detection of clinical deterioration. Further research is essential to evaluate the effectiveness across a wider range of clinical settings.

## 1 Introduction

Continuous monitoring in the Intensive Care Unit (ICU) has been crucial in enhancing the outcomes for critically ill patients (1, 2). However, this level of vigilance is often challenging to replicate in general wards due to the lack of suitable monitoring systems (3) and insufficient manpower for continuous surveillance (4). Consequently, this gap may lead to unanticipated deterioration, delayed ICU transfers, or, in severe cases, fatalities within the ward (5, 6). Early Warning Systems (EWS) have been developed and used across the world such as Modified Early Warning Score (MEWS) (7) and National Early Warning Score (NEWS) (8) to determine potential early patient deterioration in general wards. However, these tools, despite their clinical utility, are susceptible to frequent false positives, and inability to account for vital signs trends (9, 10).

Manual intermittent spot checks for heart rate (HR), respiratory rate (RR) and blood pressure (BP), conducted at intervals of 3–6 h (11, 12), are the norm for patient monitoring in general wards. However, this periodic monitoring may overlook crucial changes between checks (5, 13), consuming valuable healthcare practitioners (HCP) time (13) that could otherwise be dedicated to providing enhanced patient care. Although continuous patient contact monitoring technologies such as sphygmomanometry and oscillometery (14) for BP and electrocardiography (15, 16) for HR and RR exist, they come with drawbacks like discomfort, sleep disturbance, and skin irritation due to direct body connection (17–19). Additionally, bedside multiparameter monitoring systems with alarms can contribute to patient stress and sleep disturbance (20, 21).

**Figure 1:**
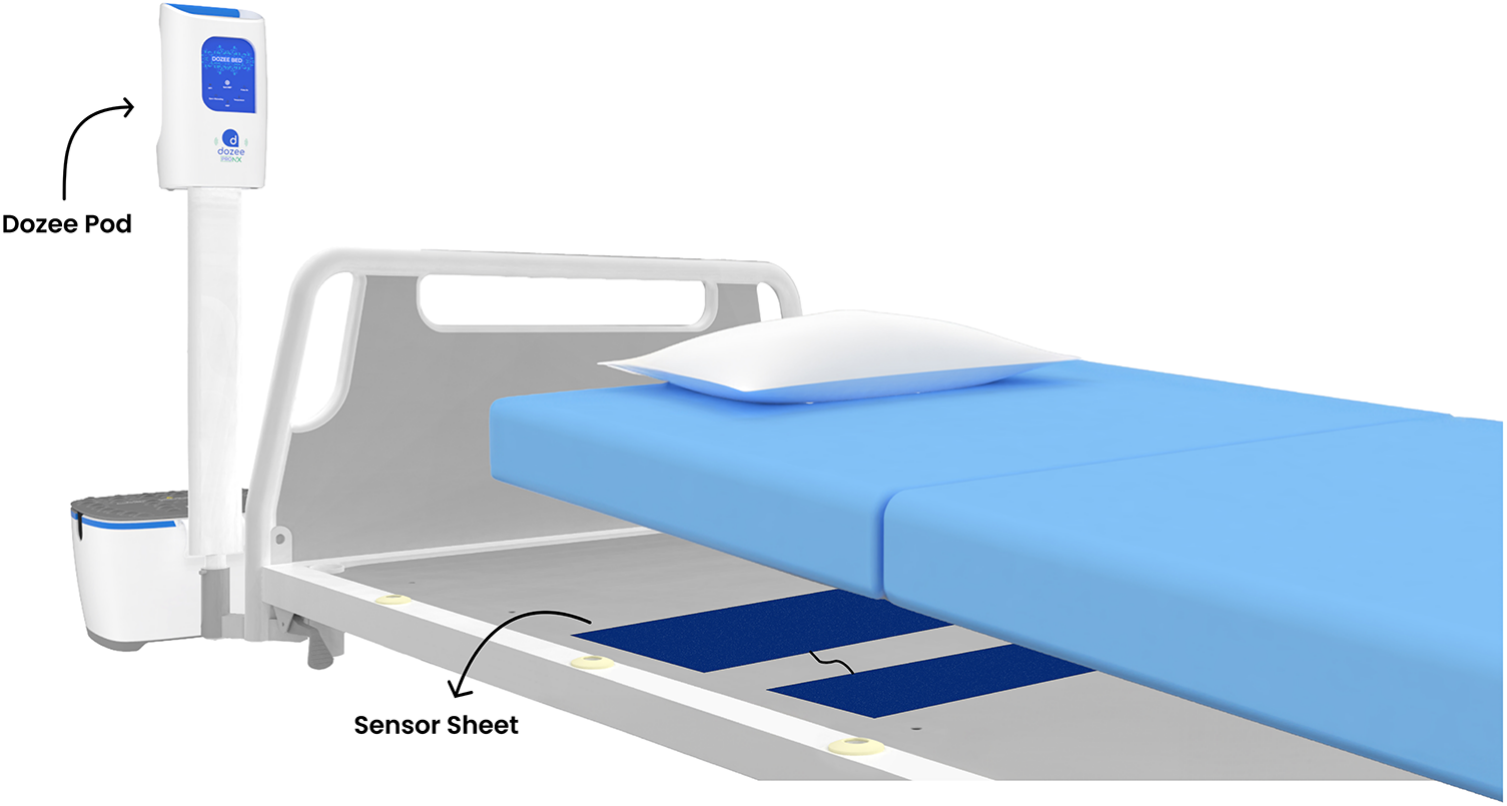
Representation of the Dozee system. Sensor sheets placed under the mattress collect ballistocardiography signals, which are transmitted to the pod and sent to the cloud for processing into vital signs data.

To address these challenges, this study introduces an innovative solution: a continuous, contactless, and remote vital signs monitoring system for HR, RR and BP utilizing ballistocardiography (Dozee system) (22–24). This system uses sensor sheets placed under the mattress to collect ballistocardiography signals, which are transmitted to the pod and then sent to the cloud for processing into vital signs data (Figure 1). Equipped with a dynamic, tier-based alerting system (Dozee-EWS) this system aims to detect early signs of patient deterioration more effectively than intermittent spot checks and contact-based monitoring. By addressing the limitations of intermittent spot checks and contact-based monitoring, Dozee-EWS has the potential to significantly reduce the incidence of clinical deterioration, delay in ICU transfers, and even fatalities in general wards. Therefore, the aim of this study was to evaluate the efficacy of the Dozee-EWS in identifying early signs of deterioration among general ward patients. Specifically, the objectives of the study were 1. To assess the potential of Dozee-EWS, in detecting clinical deterioration among patients admitted to a general ward and 2. To evaluate the HCP time spent in various activities in the wards.

## 2 Materials and methods

### 2.1 Study design

This prospective observational cohort study was conducted from October 31, 2022, to January 31, 2023, under the approval of the King George's Medical University U.P. Institutional Ethics Committee and was registered with the Clinical Trials Registry of India (CTRI/2022/10/046404). This study was done in accordance with the Declaration of Helsinki and in compliance with the requirements of Good Clinical Practice. Signed informed consent was obtained from the patients. The study solely observed patient outcomes without any clinical interventions due to Dozee-EWS. Hospital's standard-of-care was followed for care. Additionally, the study's documentation and reporting were guided by the STROBE guidelines (25).

### 2.2 Setting

The study took place at King George's Medical University (KGMU) in Lucknow, India, which is a public hospital with 4,000 inpatient beds, including 216 beds distributed across six Internal Medicine wards. The Dozee system was installed in two medicine wards with 72 beds, split equally.

### 2.3 Time-motion activity

A time-motion study was carried out over a full 24 h weekday in a designated medicine ward to assess HCP activities. This study involved closely observing a junior resident doctor who was responsible for all primary care, monitoring, and charting tasks in the ward.

### 2.4 Study participants

The study participants were patients admitted to the two medicine wards. The inclusion and exclusion criteria were:

Inclusion Criteria:

a. Adult (>18 years old) ward patients in the identified study wards
b. Weight between 40 kg and 120 kg
c. They/legal representatives are able and willing to provide written informed consent

Exclusion Criteria:

a. Admitted for observation period of <4 h
b. Weight below 40 kg and above 120 kg
c. Have any condition that could interfere with the subject's ability to lie flat or stably on the bed
d. Need for equipment whose operation can interfere with the Dozee-EWS
e. Patient not giving/unable to give consent

### 2.5 Dozee-EWS

A dynamic tier based alerting system with three tiers (Table 1) was developed. This three-tiered system is designed to prioritize alerts based on the severity of vital signs deviations, activating alerts only when significant deterioration is detected. “Snooze time,” a period without alerts, was established for three hours for Tier 1 and Tier 2. In Tier 3, there is no snooze period, and alerts are issued every 10 min. If there are no alerts during the snooze time, the system resets to the lowest threshold tier. The thresholds and snooze times for these tiers were established through clinical practice observations and inputs from clinical experts.

**Table 1:** Dozee-EWS tiered framework.

	Tier 1 range	Tier 2 range	Tier 3 range
HR (beats per minute)			
High HR	120 < HR ≤ 129	130 < HR ≤ 139	HR ≥ 140
Low HR	40 >HR ≥ 36	NA	HR ≤ 35
RR (breaths per minute)			
High RR	29 < RR ≤ 34	35 < RR ≤ 39	RR ≥ 40
Low RR	8 >RR ≥ 7	NA	RR ≤ 6
Systolic blood pressure (SysBP) (mmHg)			
High SysBP	157 < SysBP ≤ 178	NA	SysBP ≥ 179
Low SysBP	102 >SysBP ≥ 82	NA	SysBP ≤ 81

Dozee-EWS uses median values of vital signs over specific intervals—every 10 min for HR and RR, and every 30 min for BP moving with an increment of two minutes. Median values, less sensitive to outliers and artefacts, ensure alerts are triggered by sustained deviations, providing a more accurate reflection of the patient's condition. These intervals are dictated by the vital signs’ measurement frequency: HR and RR are recorded every minute, and BP every 10 min. The alerting frequency of 10 min in Tier 3 was chosen to balance timely alerts with minimizing alarm fatigue. Shorter intervals would increase the frequency of alerts, leading to alarm fatigue, while longer intervals could result in missing early signs of deterioration. This selection ensures effective early warning without overwhelming HCP.

### 2.6 Variables and data sources

HR, RR, and BP data were captured using the Dozee system, while demographic information, admission and discharge times, patient status, and length of stay (LOS) were obtained from medical records. Patients were categorized into two groups: a deterioration group (those transferred to the ICU or deceased) and a normal discharge group (those discharged under routine conditions). For both groups, the total, mean, and median number of alerts were computed at various time-points—24, 48, 72, 96, and 120 h, as well as the LOS—prior to either patient deterioration or discharge. Sensitivity and specificity were determined by classifying alerts as follows: true positives for deteriorating patients who received alerts, false negatives for deteriorating patients without alerts, true negatives for normal discharged patients without alerts, and false positives for normal discharged patients who received alerts. In this classification, no distinction is made between the different tier alerts. This means that all alerts, whether Tier 1, Tier 2, or Tier 3, are treated equally in the analysis. Additionally, the average time from the first alert to patients’ deterioration was calculated for all time periods. Time spent by HCP on various activities was also recorded over a 24 h period.

### 2.7 Bias

To mitigate selection bias, all eligible patients who met the inclusion and exclusion criteria and provided informed consent, were enrolled, without implementing any additional selection or randomization processes.

### 2.8 Study size

No formal sample size calculations were performed. The study duration was planned to enroll at least 700 patients.

### 2.9 Statistical analysis

Descriptive statistics were utilized to analyze demographic characteristics, alerts and alert timing for both study groups across all time frames. Box plots depicted the distribution of alerts between the two study groups. Descriptive statistics were again utilized to evaluate the sensitivity and specificity of alerts and the time HCP spent on various activities. To assess the normality of the continuous data, the Shapiro-Wilk test was employed. Since the continuous data did not follow a normal distribution, the Wilcoxon Rank-Sum test was performed for statistical significance. Additionally, the Chi-Square test was used for categorical variables (26). Statistical significance was defined by a *p*-value of less than 0.05 (*p* < 0.05).

## 3 Results

725 patients were admitted to the study wards and provided written consent to participate. However, two patients withdrew their consent and 17 lacked sufficient data for analysis (less than 4 h of data), leaving 706 patients for analysis. Of these, 33 experienced clinical deterioration; 30 were transferred to the ICU and 3 died. Demographic and clinical characteristics of both groups are presented in Table 2. Similarities in weight, height, and BMI were observed between the deterioration and normal discharge groups, with comparable mean ages. The deterioration group had a higher proportion of males, though not statistically significant and a statistically significant slightly shorter average LOS compared to the normal discharge group.

**Table 2:** Demographic details of the analyzed patients.

Parameters	Deterioration	Normal discharge	*P*-Value
Patients (%)	33 (5%)	673 (95%)	–
Age (years) mean; SD (Min-Max)	51; 16 (20–78)	46; 17 (18–90)	0.14
Gender (%)	19 (57%) Male; 13 (43%) Female	330 (49%) Male; 343 (51%) Female	0.25
Weight (kgs) mean; SD (Min-Max)	66; 6 (54–84)	66; 7 (40–82)	0.88
Height (cms) mean; SD (Min-Max)	157; 3 (151–161)	156; 3 (145–167)	0.21
BMI mean; SD (Min-Max)	27; 3 (21- 33)	27; 3 (17–36)	0.81
LOS (hours) mean; SD (Min-Max)	115; 132 (7–617)	119; 88 (5–455)	<0.0001*
Total monitoring time (hours)	3,832	80,616	–

SD, standard deviation; BMI, body mass index.

* Signficance.

**Figure 2:**
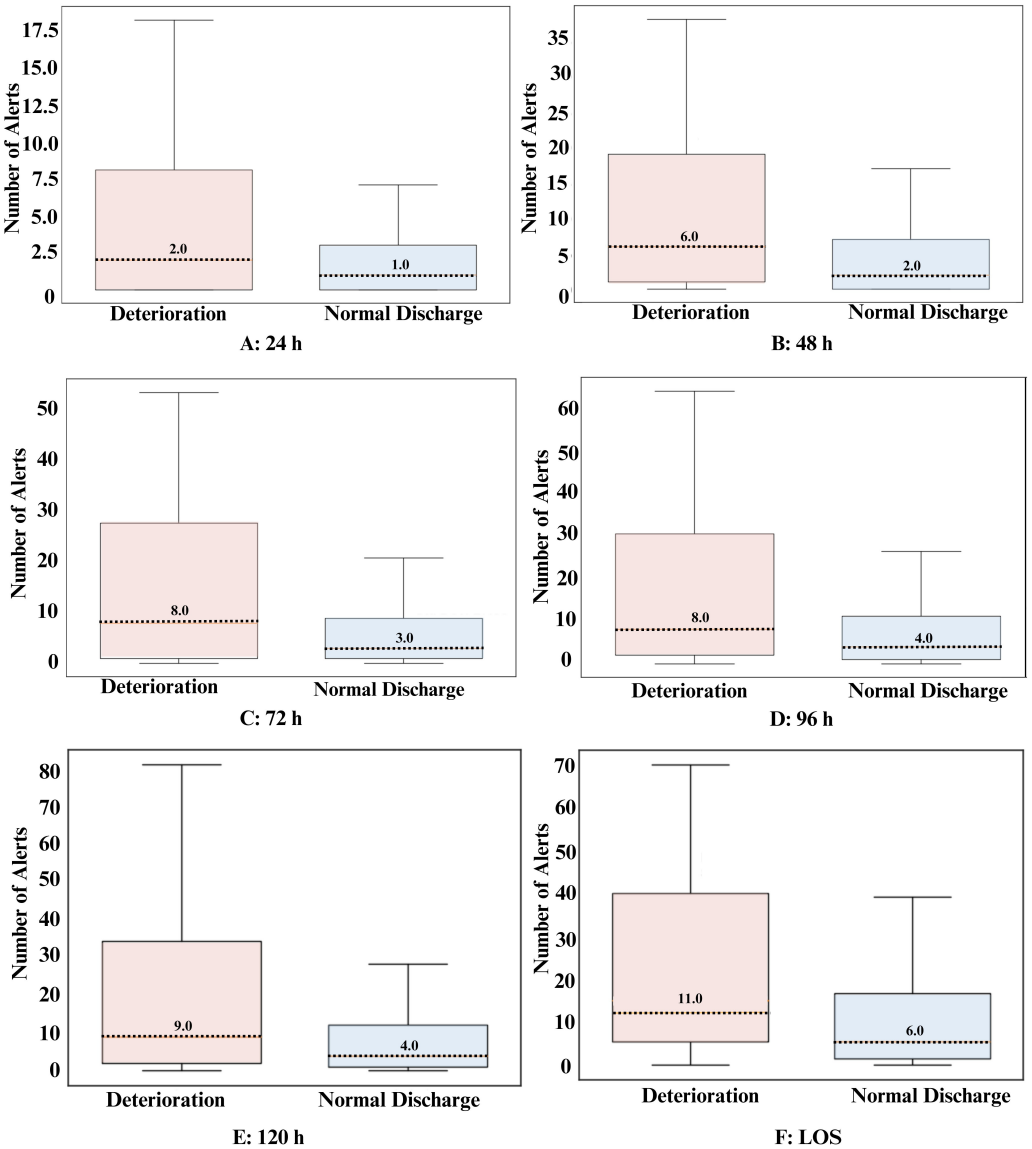
Box plot representation of the alerts for all analyzed timeframes for both groups. Dotted lines represent the median value.

All time-points demonstrated a statistically significant difference in the average number of alerts between the deterioration group and the normal discharge group, indicating the system's discriminatory ability to detect deterioration (Table 3). Across different time-points, the deterioration group consistently showed higher average alerts per patient and median alerts compared to the normal discharge group (Table 3 and Figure 2). Specifically, in the 24 h window, the deterioration group had an average of 11 alerts per patient whereas the normal discharge group had a lower average of 4 alerts per patient. This trend persisted across subsequent time-points, with the deterioration group maintaining higher average alerts per patient compared to the normal discharge group. For instance, within 48 h, the deterioration group had an average of 20 alerts per patient, while the normal discharge group had a lower average of 6 alerts per patient. In all cases, the patients who had a deterioration received an alert well in advance in the time-points analyzed. In the last 24 h the mean time of first alert to deterioration was 16 h.

**Table 3:** Alert numbers and time to first alert for all analyzed time-points for both groups.

Timeframe before event#	Group	Total alerts	Median (min-max)	Average alerts/patient Mean (SD)	*p*-value	Average time of first alert to deterioration Mean (SD)
24 h	D	384	2 (0–123)	12 (28)	0.0344*	16 (8)
	ND	2,529	1 (0–211)	4 (13)		NA
48 h	D	649	6 (0–235)	20 (45)	0.0047*	32 (18)
	ND	4,282	2 (0–228)	6 (15)		NA
72 h	D	766	8 (0–235)	23 (45)	0.0024*	44 (26)
	ND	5,791	3 (0–244)	9 (18)		NA
96 h	D	838	8 (0–235)	25 (45)	0.0036*	52 (33)
	ND	7,786	4 (0–456)	12 (29)		NA
120 h	D	909	9 (0–235)	28 (45)	0.0034*	61 (43)
	ND	9,243	4 (0–697)	14 (38)		NA
LOS	D	1,092	11 (0–235)	33 (51)	0.0059*	101 (132)
	ND	14,443	6 (0–1,329)	21 (74)		NA

D, deterioration (*n* = 33); ND, normal discharge (*n* = 676); NA, not applicable. #event-deterioration or normal discharge.

* Significance.

Across all time-points, sensitivity consistently demonstrated high values, ranging from 67% to 94%. Specificity values, on the other hand, were generally lower, ranging from 19% to 42% (Table 4).

**Table 4:** Sensitivity and specificity of Dozee-EWS for all analyzed time-points for both groups.

Timeframe before event#	True positive	True negative	False positive	False negative	Sensitivity	Specificity
24 Hours	22	284	389	11	67%	42%
48 Hours	27	204	469	6	82%	30%
72 Hours	29	164	509	4	88%	24%
96 Hours	30	146	527	3	91%	22%
120 Hours	30	139	534	3	91%	21%
LOS	31	127	546	2	94%	19%

#event-deterioration or normal discharge.

**Figure 3:**
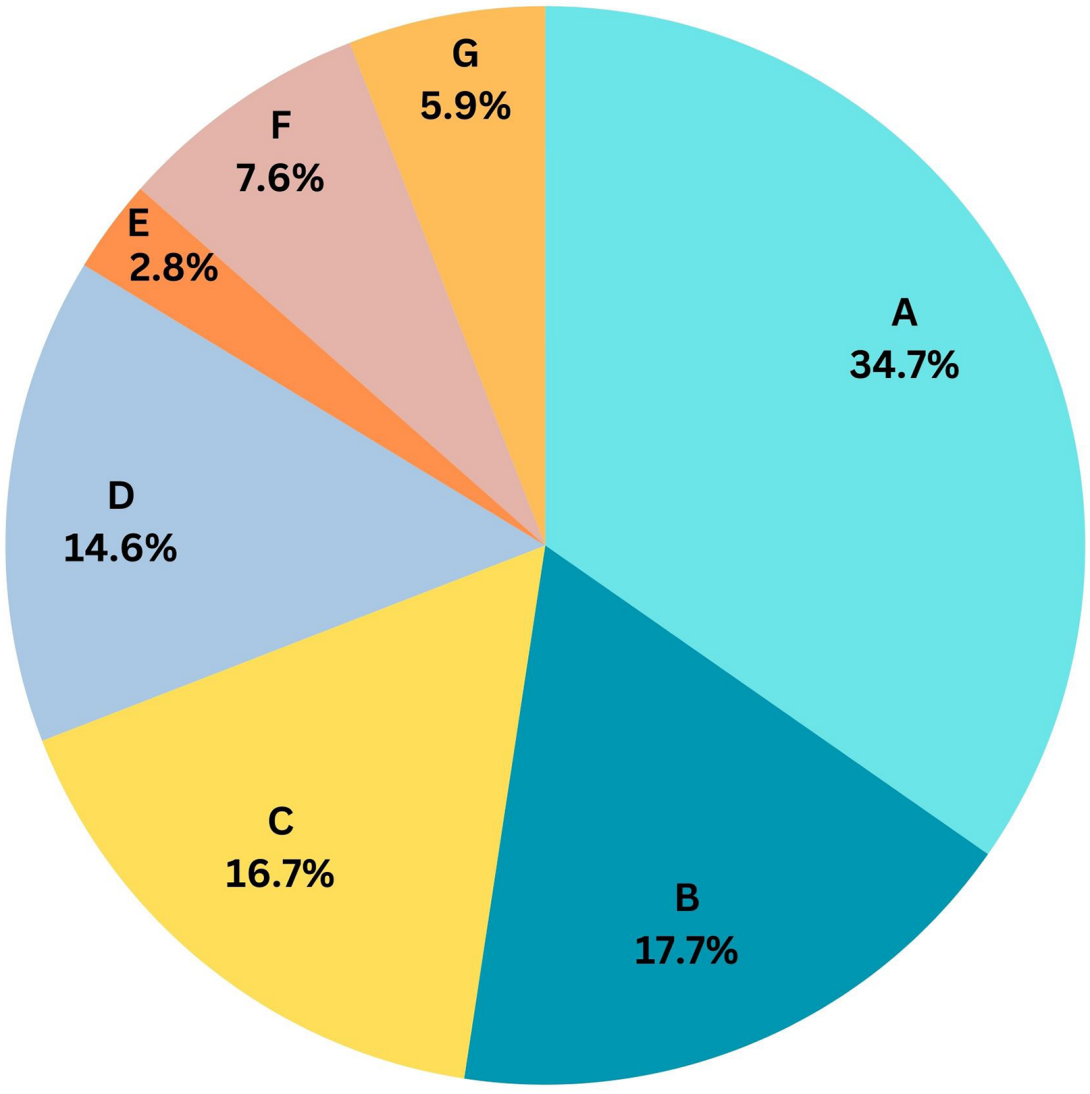
Activity wise HCP time spent in a 24 h cycle. **(A)**: Patient care, medication and mobilization (34.7%), **(B)**: Available on call/emergency (17.7%), **(C)**: Communication and coordination (16.7%), **(D)**: Other documentation (14.6%), **(E)**: Being at central station (2.8%), **(F)**: Vital checks (7.6%), **(G)**: Other activities: (5.9%).

Vitals checks and their documentation are the two primary activities, accounting for 10% of the time spent by HCP (Figure 3). The HCP-to-patient ratio was 1:36.

## 4 Discussion

The aim of this study was to evaluate the ability of the Dozee-EWS in identifying clinical deterioration signs in patients within general wards and to examine the distribution of HCP time across different ward-related activities. The results showed that the Dozee-EWS consistently produced a higher number of alerts for patients experiencing deterioration, across all timeframes. Notably, it demonstrated balanced sensitivity and specificity particularly in the critical 24 h window preceding a patient's deterioration or discharge. Additionally, the study found that HCP spent 10% of their time monitoring and documenting vital signs.

This study underscores the potential benefits of the Dozee-EWS in enhancing patient safety within the demanding environment of busy public hospital wards, particularly in resource-limited settings such as this study location (27–30). These benefits are supported by a body of literature indicating that continuous monitoring can markedly improve patient outcomes by detecting deteriorations that intermittent checks may miss (31–37).

The study groups had similar demographics except for the LOS. The deterioration group had a statistically significant shorter average LOS, which may be attributed to rapid clinical intervention following the detection of deterioration, leading to quicker transfer to the ICU.

In the critical 24 h before discharge or clinical deterioration, the Dozee-EWS proved to be effective. Patients in the deterioration group received notably more alerts—on average, 3.1 times those of the normal discharge group. Patients experiencing deterioration typically received their first alert at least 16 h before the event. This capability aligns with other established EWS like NEWS and MEWS, which are also recognized for their ability to identify at-risk individuals within a similar timeframe (7, 38–42).

The performance of the Dozee-EWS, in terms of sensitivity and specificity, was notable in the final 24 h before deterioration. For sensitivity and specificity, all alerts—whether Tier 1, 2, or 3—are treated the same. However in a real-world settings, Tier 3 alert would elicit a rapid response intervention and a Tier 1 alert would be ranked as less acute. This approach of treating all the different tier alerts ensures a consistent evaluation of the system's ability to detect clinical deterioration across all levels of severity. The sensitivity and specificity performance in this study aligns with similar studies on continuous monitoring systems despite a tendency towards lower specificity (43–45). In these studies where contact-based wearables and non-contact monitoring systems were utilized, sensitivity ranged from 55% to 74%, while specificity varied from 25% to 94% (43–45).

The potential inclusion of additional vital signs parameters such as oxygen saturation (SpO2) and temperature could further enhance sensitivity, though the existing contact-based measurement methods pose compliance challenges. Monitoring SpO2 is particularly crucial as it provides vital information about respiratory efficiency and oxygen delivery, with drops indicating potential respiratory failure requiring immediate action. Combining SpO2 with other vitals offers a comprehensive view of cardiovascular and respiratory status, enabling HCP to detect subtle changes earlier. Published research has shown that continuous SpO2 trends, in combination with other vital signs parameters, can predict clinical deterioration (43, 46).

The Dozee system features a pod that easily connects to wired or wireless SpO2 and temperature sensors. These plug-and-play accessories allow effortless attachment or removal without changes to the existing setup, enabling seamless system expansion to meet specific patient needs. In line with existing tiered alerts for HR, RR, and BP, tiered alerts for SpO2 and temperature can be integrated into the framework. This can ensure prompt detection of significant deviations, enhancing the system's overall performance in monitoring patient health.

The prevalence of false alerts, indicated by lower specificity, raises concerns about alarm fatigue among HCP, underscoring the need for a careful balance in the system's alert settings (47, 48). The effectiveness of an EWS hinges on its alert-triggering rules; missing many deteriorating patients or over-alerting in normal cases is impractical and does not meet clinical standards.

Additionally, the study analysis highlighted potential time management efficiencies for HCP. Given that the HCP-to-patient ratio at this study site was four times higher than recommended norms (4), the automation facilitated by this system could save about 140 min per HCP each day. This finding is supported by an independent report from Sattva Consultants, which indicates that such systems could reduce nursing time by up to 2.5 h daily (49). Given many countries’ low nurse-patient ratio and the public healthcare system's resource constraints, this system can allow an HCP to monitor more patients per day and focus more time on direct care activities.

This study's strength is its innovative deployment of continuous, contactless monitoring technology across a large cohort, totalling over 84,448 h of monitoring, which has the potential to significantly enhance patient safety in general wards. The ability of the Dozee-EWS to detect deterioration early is of paramount importance, potentially allowing HCP to intervene sooner and more effectively. Clinically, this technology can be instrumental in bridging the monitoring gap in ward settings, where continuous surveillance has traditionally been limited. Implementing such technology may reduce the need for frequent physical checks, thus optimizing HCP resources and enhancing overall patient care.

Despite its contributions, the study is limited by its single-center design and observational nature, which restricts the ability to generalize findings. Additionally, the variability in patient comorbidities and clinical conditions, which were not fully explored, may affect the applicability of the results in other settings. Further research is warranted to assess the effectiveness in a broader array of clinical environments and to consider integrating more comprehensive vital signs parameters to refine its sensitivity and specificity.

## 5 Conclusion

The implementation of the Dozee-EWS in a general ward setting demonstrates benefits in enhancing patient safety through timely alarm that can facilitate early detection of deterioration. Additionally, this system can offer significant time savings, allowing HCP to optimize care and focus more on direct patient interactions. Despite the limitations of its single-center and observational design, these findings highlight the potential of advanced monitoring technologies to improve clinical outcomes and operational efficiency in resource-constrained hospital settings. Future research should aim to expand these results across diverse environments and incorporate additional vital signs parameters to maximize the system's clinical utility.

## Data Availability

The raw data supporting the conclusions of this article will be made available by the authors, without undue reservation.
